# Are Biogenic and Pyrogenic Mesoporous SiO_2_ Nanoparticles Safe for Normal Cells?

**DOI:** 10.3390/molecules26051427

**Published:** 2021-03-06

**Authors:** Katarzyna Solarska-Ściuk, Kinga Adach, Sylwia Cyboran-Mikołajczyk, Dorota Bonarska-Kujawa, Agnieszka Rusak, Łucja Cwynar-Zając, Tomasz Machałowski, Teofil Jesionowski, Katarzyna Grzywacz, Mateusz Fijałkowski

**Affiliations:** 1 Department of Physics and Biophysics, Wrocław University of Environmental and Life Sciences, 50-375 Wrocław, Poland; sylwia.cyboran@upwr.edu.pl (S.C.-M.); dorota.bonarska-kujawa@upwr.edu.pl (D.B.-K.); katarzynag448@gmail.com (K.G.); 2 Department of Advanced Materials, Institute for Nanomaterials, Advanced Technologies and Innovation, Technical University of Liberec, 461 17 Liberec, Czech Republic; Kinga.Adach@tul.cz (K.A.); mateusz.fijalkowski@tul.cz (M.F.); 3 Department of Histology and Embryology, Medical University of Wrocław, 50-367 Wrocław, Poland; rusakagn@gmail.com (A.R.); lucja.cwynar-zajac@umed.wroc.pl (Ł.C.-Z.); 4 Institute of Chemical Technology and Engineering, Faculty of Chemical Technology, Poznan University of Technology, 60-965 Poznań, Poland; tomasz.g.machalowski@doctorate.put.poznan.pl (T.M.); teofil.jesionowski@put.poznan.pl (T.J.)

**Keywords:** mesoporous silica, biogenic and pyrogenic silicon dioxide, nanoparticles, cytotoxicity

## Abstract

Silicon dioxide, in the form of nanoparticles, possesses unique physicochemical properties (size, shape, and a large surface to volume ratio). Therefore, it is one of the most promising materials used in biomedicine. In this paper, we compare the biological effects of both mesoporous silica nanoparticles extracted from *Urtica dioica L.* and pyrogenic material. Both SEM and TEM investigations confirmed the size range of tested nanoparticles was between 6 and 20 nanometers and their amorphous structure. The cytotoxic activity of the compounds and intracellular ROS were determined in relation to cells HMEC-1 and erythrocytes. The cytotoxic effects of SiO_2_ NPs were determined after exposure to different concentrations and three periods of incubation. The same effects for endothelial cells were tested under the same range of concentrations but after 2 and 24 h of exposure to erythrocytes. The cell viability was measured using spectrophotometric and fluorimetric assays, and the impact of the nanoparticles on the level of intracellular ROS. The obtained results indicated that *bio*SiO_2_ NPs, present higher toxicity than pyrogenic NPs and have a higher influence on ROS production. Mesoporous silica nanoparticles show good hemocompatibility but after a 24 h incubation of erythrocytes with silica, the increase in hemolysis process, the decrease in osmotic resistance of red blood cells, and shape of erythrocytes changed were observed.

## 1. Introduction

Nanotechnology is a modern field of science focused on designing, manufacturing and applying of a wide range of various types of particles, ranging in size from several to several dozen nanometers. Moreover, this knowledge is useful in virtually every area of life and, what is important, has practical implications in biology and medicine. From a broad range of nanoparticles which has been studied in recent years, the silicon dioxide nanoparticles (SiNPs—whose size ranges between 5–1000 nm) draw particular attention. They are characterized by a low density and toxicity as well as biocompatibility and biodegradability. One example of SiO_2_ nanparticles are mesoporous silica nanoparticles (MSNs), which possess a large surface area and pore volume, both properties which make them most promising drug delivery vehicles. They also have high adsorption capacity which ensures encapsulation. Furthermore, they have a unique optical and a specific surface and this ensures their wide use [1,2]. The collected data demonstrates a high level of in vivo biocompatibility of both types of silica nanoparticles, before and after surface functionalization. Many in vitro studies show that SiO_2_ at doses of up to 100 μg/mL is non-toxic for cells, but at higher doses, it induces a cytotoxic effect [2,3]. Mesoporous silica nanoparticles are perfect for biomedical applications. The unique possibilities of functionalization, the biocompatibility of nanoparticles, and their small size, which enable them to a non-invasive insertion of the material into the living cells, make them widely applicable, e.g., in gene transfection reagents, cell markers, and molecule carriers. What is more, modified silica nanoparticles play a dual role, namely they can to recognize the tumor cells and subsequently, at appropriate intervals, they also release the antineoplastic drugs [4,5]. The biocompatibility of silica nanoparticles is a subject of a serious academic dispute, as studies conducted by researchers led to variable results. Despite these controversies, it is worth noting that these nanoparticles, based on the encouraging results of pre-clinical studies, received approval from the Food and Drug Administration (FDA) as an Investigational New Drug (IND) to conduct a phase I human clinical trial [6].

Several different methods used to obtain silica nanoparticles are known. One of them is green synthesis, which attracts great attention in the areas of biology, medicine, pharmaceutical, and other fields of science. Typically, these nanoparticles are synthesized using different chemical and physical methods (such as chemical vapour condensation, flame synthesis, or laser ablation) which are complex and very expensive. Moreover, the above methods may contribute to potential environmental and biological hazards. In contrast, green synthesis of nanoparticles based on using plants is highly efficient, fast, gen low cost and eco-friendly [7]. In recent years a large group of scientists has adopted green synthesis methods for the production of nanoparticles, including calcium, copper, gold, iron, silica, silver or zinc from different medicinal plants [8]. In this paper, we present a chemical method that was used to remove the organic part of the plant (cellulose, hemicellulose, lignin) and an evaluation of biological effects regarding amorphous SiO_2_ extracted from *Urtica dioica L*. Additionally, the results obtained from a study focused on pyrogenic, namely commercially available silica nanoparticles, are compared.

Stinging nettle (*Urtica dioica L.*) is a well-known plant species. This crop (while considered as a plant produced in agriculture) is attractive for both scientifically and commercially concentrated studies, as it is the source of many added-value, natural products which are obtained from all parts of the plant, namely stem, leaves, roots or seeds. Moreover, this plant is a good source of vitamins, minerals (e.g., iron, calcium, potassium, and silica), fiber, protein and, in particular, it also has biologically active compounds which show antioxidant properties [9,10,11]. Furthermore, stinging nettle contains silicon at the concentration of 1.75% by mass [12]. Silicon is a common element on Earth. It is present in most cells and is also necessary for growth and bone calcification. Silicon is also necessary for human health due to the fact that it has beneficial effects and cures several human disorders, including osteoporosis, aging of the skin, hair, and nails as well as atherosclerosis. It is believed that silicon may also have anticancer, antiatherosclerotic, and antidiabetic effects. This element is present in all tissues of the body and, what is noteworthy, its content clearly decreases with age, a factor which is responsible for the progress of degenerative processes in the human body [13].

In the present manuscript, a novel method of producing high-purity biogenic silica nanoparticles (*bio*SiO_2_) from stinging nettle and of analyzing their biocompatible properties using in vitro cell-based research tools is proposed. The high purity of the obtained material was confirmed by ATR FTIR analysis. The biogenic silica nanoparticles were also well characterized by SEM and TEM investigations which confirmed the size of the tested mesoporous silica (6 and 20 nanometers). Furthermore, the biocompatibility of the silica nanomaterials was evaluated at the cellular levels using spectrophotometric (MTT) and fluorimetric (Hoechst 33258) assays. Moreover, the impact of the nanoparticles on the level of intracellular reactive oxygen species (ROS) was assessed. Similarly, the pyrogenic silica (*pyr*SiO_2_, synthetically produced, purchased from Sigma Aldrich) was tested using the same methods and the results were compared with *bio*SiO_2_. In these studies, we used immortalized human microvascular endothelial cells (HMEC-1) and erythrocytes. Whereas in a large number of papers the data is collected on the basis of tests focused on cancer cells, the effects exerted on normal cells are not discussed. This is an interesting observation which we would like to point out, namely the fact that although silica nanoparticles which are extracted from *Urtica dioica L.* present slightly higher toxicity than *pyr*SiO_2_ nanoparticles in immortalized human microvascular endothelial cells (HMEC-1), red blood cells showing higher concentration of *pyr*SiO_2_ are more toxic than *bio*SiO_2_. What is more, the shape of erythrocytes changed after treating them with red blood cells with SiO_2_. This data proves that biogenic nanoparticles are safe when added to the normal cells, but only in the appropriate dosage. They show good compatibility and are highly promising for future studies for drug transporters.

## 2. Results

### 2.1. Surface and Morphological Properties

**Figure 1:**
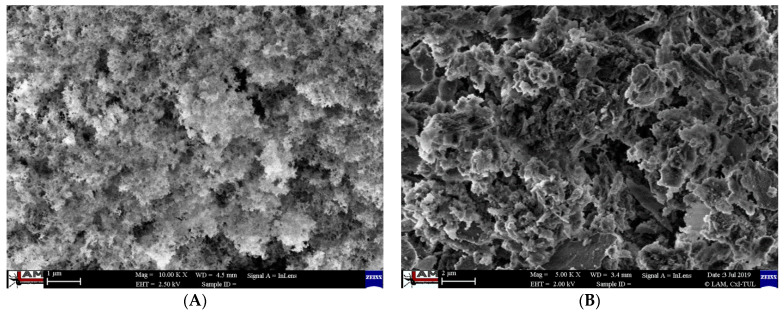

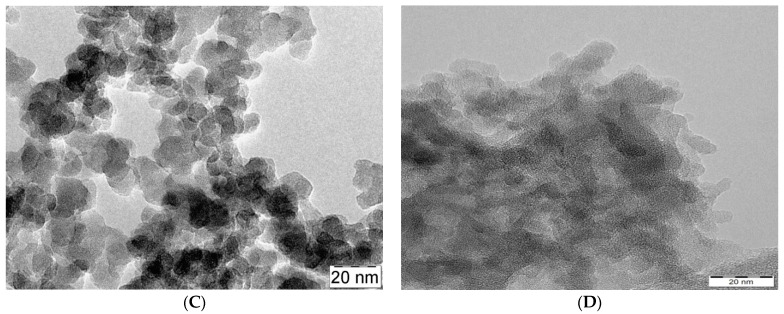
Characterization of silica nanoparticles. Silicon dioxide NPs ((**A**)—*pyr*SiO_2_ and (**B**)—*bio*SiO_2_) were characterized by a scanning electron microscope (SEM). Analysis with SEM indicates that SiO_2_ nanoparticles in form of the dried powders have a tendency to form agglomerate structures. Transmission electron microscopy (TEM) ((**C**)—*pyr*SiO_2_ and (**D**)—*bio*SiO_2_) was used to determine the shape and size of the nanoparticles.

One of the purposes of this article was to evaluate and compare the biological effects of both types of tested silica nanoparticles. Transmission and scanning electron microscopy methods were used to determine the size and exact shape of the nanoparticles as well as their form. Analyses showed that the silicon dioxide nanoparticles obtained from stinging nettle have a tendency to form agglomerates in aqueous dispersions. Whereas the size of these agglomerates is greater than a dozen nanometers, a basic form of each nanoparticle has an oval shape with size within the range of 6–20 nm (Figure 1).

### 2.2. Chemical Functionality, Dispersion and Thermal Stability Properties

**Figure 2:**
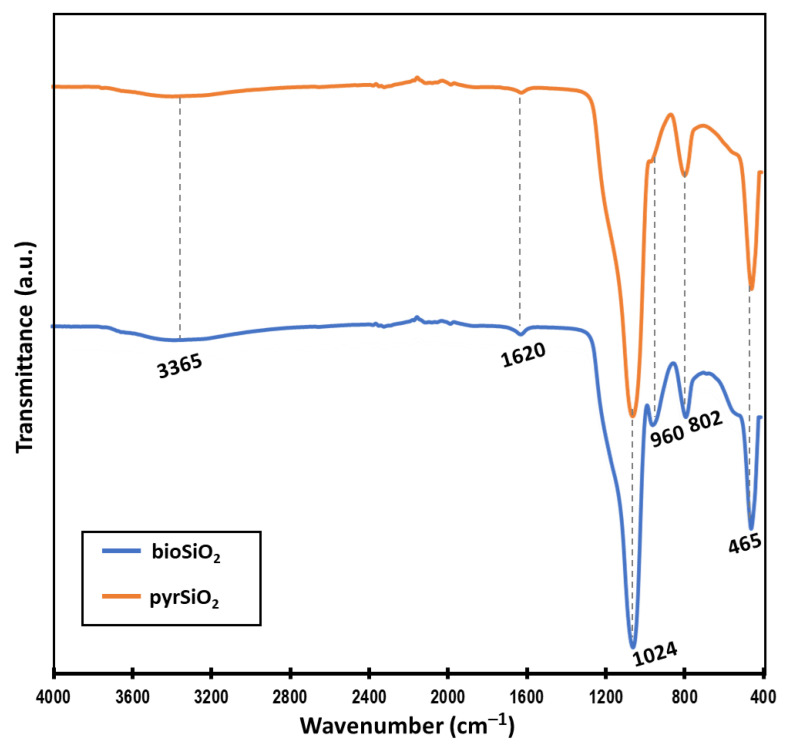
ATR-FTIR spectra of biogenic silica nanoparticles (*bio*SiO_2_ blue line) and standard silica (*pyr*SiO_2_ orange line). Strong similarity of both spectra indicates the effectiveness of the biogenic silica isolation method.

The first visible deflection of the spectra visible in Figure 2 may be observed at 3365 cm^−1^ and corresponds to H–O–H stretching vibrations (absorbed water) and H–O–H bending vibrations of water molecules, respectively [14,15]. Then, the most intensive band with a maximum near to 1024 cm^−1^ unambiguously represents the Si-O and Si-O-Si bonds characteristic for silica [15,16,17]. The peak detected at 960 cm^–1^ visible only into biogenic silica nanoparticles (*bio*SiO_2_) spectra is related to Si–O stretching vibrations of Si–OH groups and usually corresponded with amorphous silica framework [18] or biogenic based silica [19]. The absorption bands at 802 and 465 cm^−1^ are characteristic for symmetric stretching vibrations of Si–O–Si bonds and Si–O bending vibrations of siloxane groups, respectively [18,20]. Furthermore, in both spectra peaks at 1620 cm^–1^ are present. There was estimated that this signal is assigned to the bending vibrations of water [21,22].

**Figure 3:**
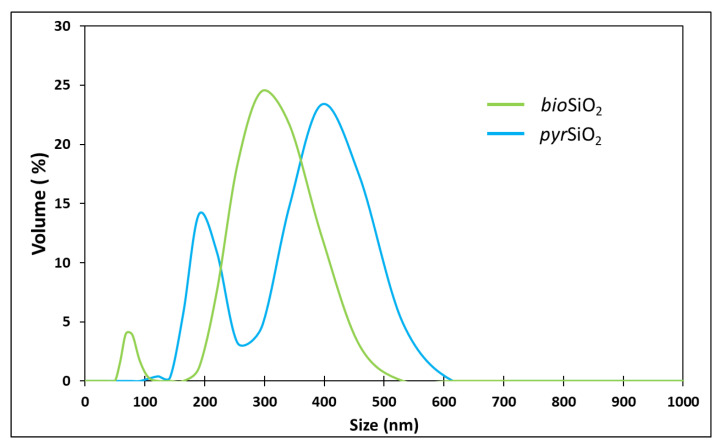
Particle diameters size distributions of *bio*SiO_2_ (green line) and *pyr*SiO_2_ (blue line) powders.

The particle size distribution of the *pyr*SiO_2_ and *bio*SiO_2_ powder materials was analysed (see Figure 3). The results show that both samples represent split graphs of two size fractions, with a smaller and a much greater particle diameter, respectively. In the case of biogenic silica, the volume fraction of smaller particles displayed diameters in the 60–105 nm range, whereas pyrogenic silica possesses a 160–250 nm range fraction. Broad bands of larger particles corresponding to diameters in the 260–610 nm (*bio*SiO_2_) and 190–530 nm (*bio*SiO_2_) range have been also observed. The observations of such particle size distributions may be associated with the formation of silica aggregate structures [23], in agreement with the TEM analysis results (see Figure S1). It should be highlighted that the volume fraction of particles having the dominant diameter in each case does not exceed 25%.

**Figure 4:**
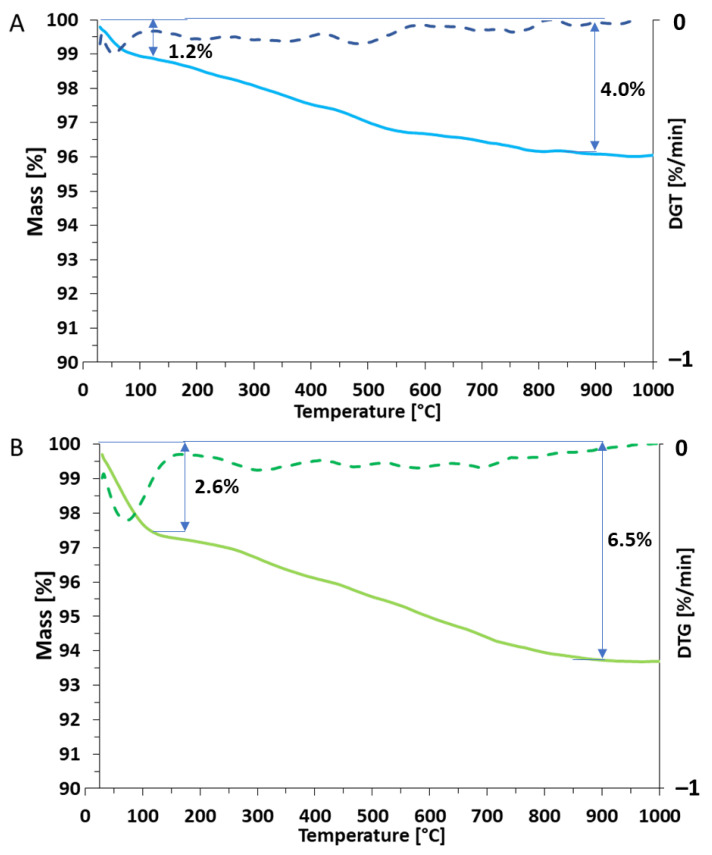
Results of thermogravimetric analysis TGA/DTG of both samples *pyr*SiO_2_ (**A**) and *bio*SiO_2_ (**B**).

Figure 4 shows the thermogravimetric curves for both analyzed silica types. In both cases the TGA curves exhibit slow decreases as the temperature increases during the TGA analysis, what is characteristic for silica [24]. For *pyr*SiO_2_ mass loss at 130 °C is approximately 1.2%, while in *bio*SiO_2_ this value increased to 2.6%. This could be related to residual moisture desorption and the evaporation of the water molecules bound within the samples [25]. A plateau covering the temperature range over 850 °C indicates unchanged weight, i.e., a thermally stable product was obtained. Final decomposition of materials has been estimated at 4% for pyrogenic and 6.5% for biogenic silica, respectively. Such an observation may indicate the presence of a low organic content and its decomposition into biogenic silica and should be confirmed in future research.

### 2.3. The Microscopic Studies of HMEC-1 Shapes

Morphological changes in HMEC-1 cells were analyzed after treating them with silica nanoparticles (biogenic and pyrogenic) at the appropriate concentration (0, 20, 50, 100, and 200 μg/mL) and after three periods of incubation. When the concentration of nanoparticles was higher, both types of changes, namely morphological and in size were observed. Similarly, when the concentration and incubation in time increased, the number of cells decreased. It was noticed that the size of control cells after 72 h incubation is 33.48 ± 1.1 μm but for the concentration of 50 μg/mL is 25.63 ± 1.3 μm (*pyr*SiO_2_) and 25.78 ± 0.9 μm for *bio*SiO_2_. With the dosages (20, 50, 100, and 200 mg/mL) increasing, general tendency was the same for both type of silica nanoparticles.

**Figure 5:**
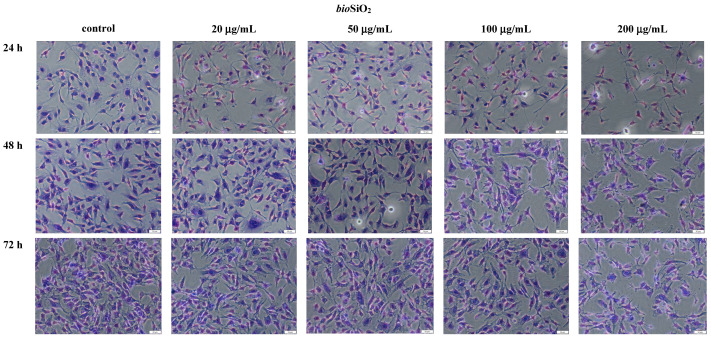
Morphological changes of the cells HMEC-1 before and after treatment with silica nanoparticles (biogenic) at concentration of 0–200 μg/mL and after three periods of incubation. The scale bar corresponds to 50 μm.

**Figure 6:**
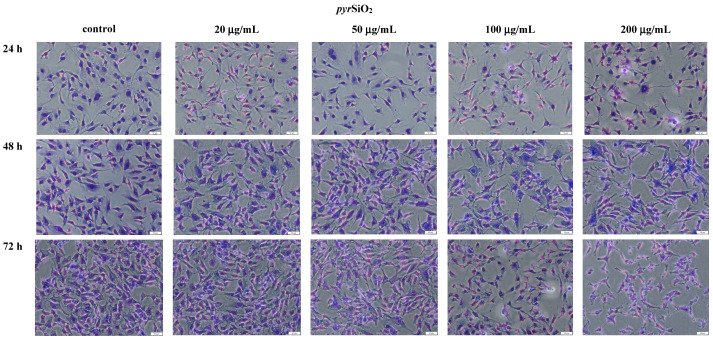
Morphological changes of the cells HMEC-1 before and after treatment with silica nanoparticles (pyrogenic) at concentration of 0–200 μg/mL and after three periods of incubation. The scale bar corresponds to 50 μm.

The morphological changes of HMEC- 1 became more and more obvious. What is more, cell density reduction, irregular shape and cellular shrinkage were observed (Figure 5 and Figure 6). Compared with the control group, the cell density in the 100 μg/mL and 200 μg/mL treated groups was obviously reduced after 24, 48 and 72 h exposure. It was found that the cellular morphology changes were directly reflected with the cell viability.

### 2.4. MTT Test and DNA Content

**Figure 7:**
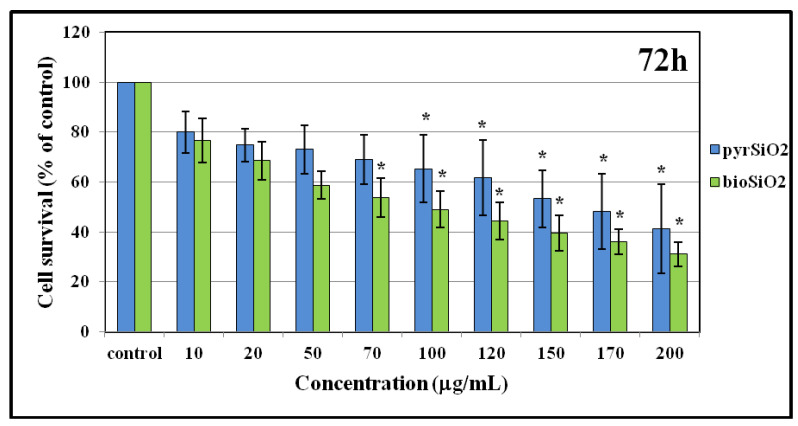
Cytotoxicity of HMEC-1 induced by silica nanoparticles. Cell survival of human dermal microvascular endothelial cell line of silica nanoparticles (pyrogenic and biogenic) was measured by MTT assay after 72 h exposure. Statistical evaluation of differences was made using the ANOVA I and Tukey’s post hoc test, at significance levels of *p* < 0.01 (*) with respect to control.

**Figure 8:**
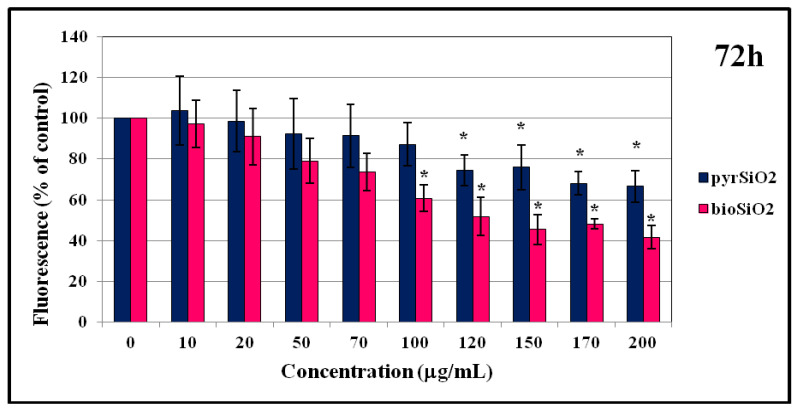
Assay of cell survival based on DNA content. Cell survival of HMEC-1 cell line of silica nanoparticles (pyrogenic and biogenic) was measured by Hoechst 33258 assay after 72 h exposure. Statistical evaluation of differences was made using the ANOVA I and Tukey’s post hoc test, at significance levels of *p* < 0.01 (*) with respect to control.

Cell viability and survival after treatment with silica nanoparticles obtained by a different methods (*pyr*SiO_2_ and *bio*SiO2) were analyzed. Silica nanoparticles obtained from stinging nettle present higher toxicity in the MTT test (at a concentration above 50 μg/mL) than pyrogenic NPs in immortalized human microvascular endothelial cells (HMEC-1) (Figure 7). The results obtained by MTT assay were confirmed using the fluorimetric method (Hoechst 33258) (Figure 8) which intercalates into DNA and makes it possible to analyze the viability and survival of cells on the basis of DNA content. Biogenic and pyrogenic silica nanoparticles at the concentrations of 50 μg/mL decreased the survival of HMEC-1 cells to about 80% when compared to control. In the Hoechst test, the maximal decrease of cell survival was observed for the highest concentration i.e., 200 μg/mL (30–40% for *bio*SiO_2_). It is worth noting that significant statistical differences in cell survival rate between *pyr*SiO_2_ and *bio*SiO_2_ nanoparticles were not observed.

### 2.5. ROS Production

**Figure 9:**
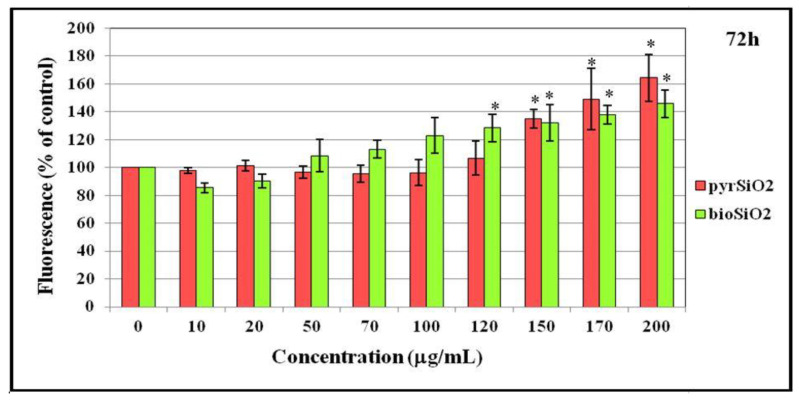
Intracellular reactive oxygen spices (ROS) generation in HMEC-1 cells. Production of ROS of human dermal microvascular endothelial cell line after treatment of silica nanoparticles (pyrogenic and biogenic). Statistical evaluation of differences was made using the ANOVA I and Tukey’s post hoc test, at significance levels of *p* < 0.01 (*) with respect to control.

Silica nanoparticles both *pyr*SiO_2_ and *bio*SiO_2_ enhanced reactive oxygen species production in endothelial cells (HMEC-1) after 24, 48, and 72 h incubation. What is more, the level of ROS is concentration-dependent on silica. If the concentration of nanoparticles is higher, the level of reactive oxygen species increases. It is particularly visible above the concentration of 100 μg/mL in all incubation periods. The results demonstrated that silica nanoparticles induced intracellular reactive oxygen species generation that increased in a dose-dependent manner. It is worth noting that significant statistical differences at the level of ROS between *pyr*SiO_2_ and *bio*SiO_2_ nanoparticles were not observed (Figure 9).

### 2.6. Hemolysis and Osmotic Resistance of Erythrocytes

**Figure 10:**
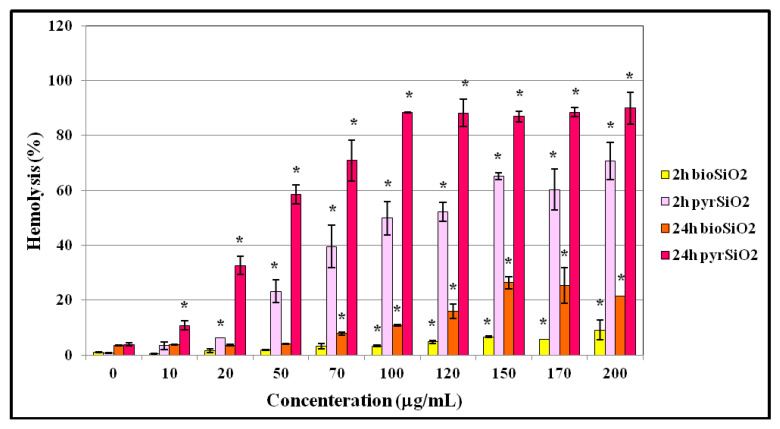
Percentage of hemolysis of erythrocytes. The hemolytic toxicity of *bio*SiO_2_ and *pyr*SiO_2_ nanoparticles concerning red blood cells was analyzed after 2 h and 24 h of incubation. Statistical evaluation of differences was made using the ANOVA I and Tukey’s post hoc test, at significance levels of *p* < 0.01 (*) with respect to control.

The hemolytic toxicity of *bio*SiO_2_ and *pyr*SiO_2_ nanoparticles in relation to red blood cells was analyzed. The control indicated that hemolysis reached 0.82% (after 2 h of incubation) and 3.7% (after 24 h of incubation) (Figure 10). Within the range of the used concentrations (0–200 µg/mL), the *pyr*SiO_2_ nanoparticles showed hemolytic toxicity and relation between hemolysis and the highest concentration equals 70.75% after 2 h of incubation, and 90.05% after 24 h of incubation. It means that in these samples a vast majority of red blood cells were hemolyzed. The obtained results show that the tested *pyr*SiO_2_ nanoparticles generate hemolysis which is both concentration and time-dependent. When the concentration of *pyr*SiO_2_ nanoparticles increases, the observed level of hemolysis increases, an observation which was proved after 2 and 24 h of interaction between nanoparticles and red blood cells. Moreover, it was observed that after 24 h of incubation, the level of hemolysis is significantly higher than after 2 h of incubation for the same concentrations. Furthermore, extremely interesting results were obtained in relation to *bio*SiO_2_ nanoparticles, for which the level of hemolysis after 2 h of incubation equals 9.09% and after 24 h of incubation 21.44%. When compared to *pyr*SiO_2,_ these nanoparticles are much safer for erythrocytes and non-toxic in the highest concentrations. Moreover, significant differences between biogenic and pyrogenic silica nanoparticles were noticed.

**Figure 11:**
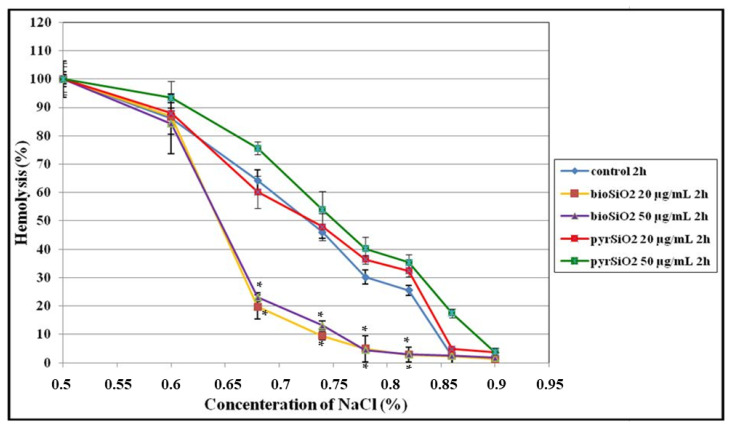

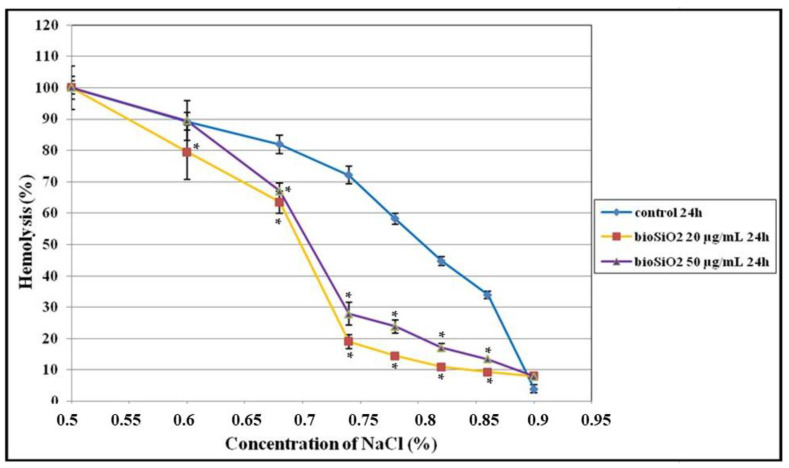
Percentage of hemolysis of erythrocytes. The osmotic resistance of red blood cells modified by SiO_2_ nanoparticles after 2 h and 24 h of incubation. Statistical evaluation of differences was made using the ANOVA I and Tukey’s post hoc test, at significance levels of *p* < 0.01 (*) with respect to control.2.7. The microscopic studies of erythrocytes shapes.

In another experiment, we tested the osmotic resistance of red blood cells modified by SiO_2_ nanoparticles (pyrogenic and biogenic). Based on the previous study, which was focused on hemolytic toxicity of nanoparticles, in this experiment, two non-lytic concentrations, namely 20 µg/mL and 50 µg/mL were used. Figure 11 presents the curves of osmotic resistance observed in erythrocytes which were modified by given nanoparticles. On the basis of the obtained data, one can identify this level of osmotic resistance in relation to the concentration of psychological saline (IC_50_) which is responsible for 50% hemolysis of erythrocytes which were modified by SiO_2_ nanoparticles (Table 1). The obtained results show that 50% hemolysis of erythrocytes treated with *pyr*SiO_2_ (50 µg/mL) nanoparticles is observed at the concentration of 0.72% NaCl (after 2 h of incubation). In relation to *bio*SiO_2_ (50 µg/mL), the results are as follows: 0.63% NaCl (after 2 h of incubation) and at the concentration of 0.65% (after 24 h of incubation).

The obtained results show that *pyr*SiO_2_ nanoparticles at a concentration of 50 µg/mL after 2 h of incubation with RBCs are responsible for a small decrease in osmotic resistance and, consequently, an increase in the level of hemolysis. In contrast, the results obtained for *bio*SiO_2_ show that the resistance of erythrocytes increased, what indicates a shift in the hemolytic curve towards lower NaCl concentrations (Figure 11). This, in turn, is reflected in a lower level of hemolysis in relation to the control sample. It means that biogenic nanoparticles which are obtained from nettle are safe for red blood cells.

**Figure 12:**
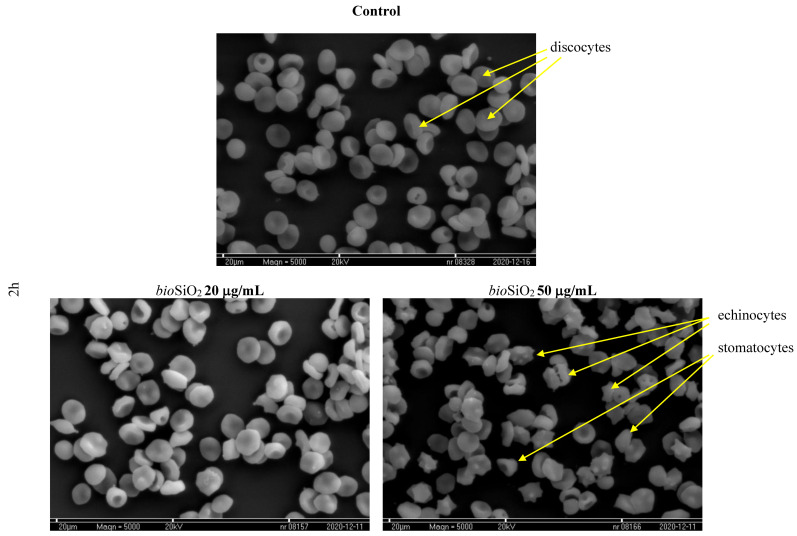

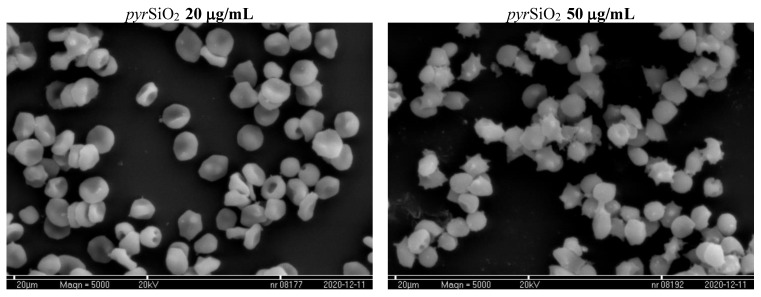
Change of erythrocyte’s shape after treatment red blood cells with different concentration of SiO_2_ and 2 h incubation: control, *bio*SiO_2_ 20 µg/mL, *bio*SiO_2_ 50 µg/mL, *pyr*SiO_2_ 20 µg/mL, *pyr*SiO_2_ 50 µg/mL. The scale bar corresponds to 20 μm.

The impact of SiO_2_ nanoparticles on the shape of red blood cells was determined using a microscope. On the basis of the photographs taken, the changes caused by the presence of the analyzed compounds were investigated. The obtained results prove that SiO_2_ nanoparticles are responsible for changes in the shape of erythrocytes. Selected photographs, which were taken using a scanning electron microscope, are presented in Figure 12. As it is presented in relation to the control sample, a vast majority of cells were of regular shapes (discocytes). In the presence of *pyr*SiO_2_ nanoparticles in the concentration of 20 μg/mL (after 2 h of incubation), echinocytes were observed, but still, the predominant group was formed by discocytes. In case of the concentration of 50 μg/mL, much more echinocytes were observed. Moreover, it was also noticed that under the concentration of 50 μg/mL erythrocytes show a tendency to aggregate and to form clumps of cells. Both selected concentrations of mesoporous SiO_2_ nanoparticles induce the shape of echinocytes and stomatocytes.

Furthermore, in the case of *pyr*SiO_2_ nanoparticles, the number of echinocytes and stomatocytes was higher than in case of *bio*SiO_2_ in both concentrations. When the period of incubation was elongated to 24 h, an increase in the number of red blood cells in the shape of echinocytes and stomatocytes for *bio*SiO_2_ was observed. Due to the toxicity of *pry*SiO_2_ in relation to RBC, a microscopic examination for incubation time of 24 h was not performed.

**Figure 13:**
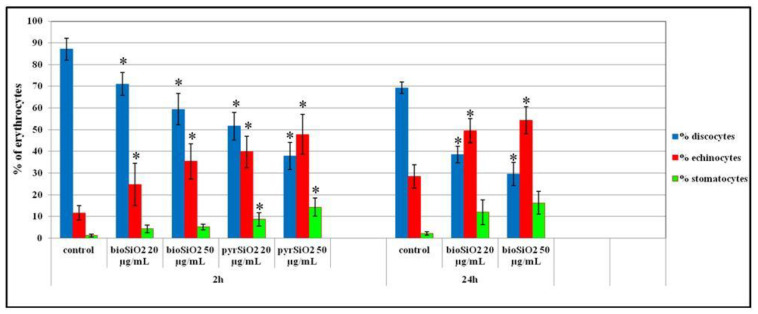
The microscopic studies of erythrocytes shapes. The results prove that SiO_2_ nanoparticles are responsible for changes in the shape of erythrocytes after 2 h and 24 h of exposure. Statistical evaluation of differences was made using the ANOVA I and Tukey’s post hoc test, at significance levels of *p* < 0.01 (*) with respect to control.

Referring to *pyr*SiO_2_ and under a concentration of 50 μg/mL, the predominant number of red blood cells was in the shape of stomatocytes and echinocytes. This is plausibly connected with the concentration of the implemented nanoparticles in the outer layer of the lipid bilayer which leads to an increase in its size in relation to the inner layer and causes changes in the curvature of the bilayer. This, in turn, leads to the deformation of the membrane and irregularities (Figure 13).

## 3. Discussion

Silica-based nanoparticles have been broadly used in various areas of biomedicine and biotechnology. Nanosilica is a highly promising material which is used in drug delivery, gene therapy, biomolecule detection, bioimaging and photodynamic therapy [26]. As a consequence of their extremely high potential, mesoporous silica nanoparticles are intensively studied with respect to different types of cells. It is crucially important to understand the interaction between SiNPs and endothelial cells, as they can be directly exposed to nanoparticles. When injected into the body, silica nanoparticles can directly contact with endothelial cells. It is a well-known fact that a single layer of endothelial cells, which lines the lumen of all blood vessels, is not only a barrier between circulating blood and a vessel wall but also is a key factor responsible for the maintenance of vascular function and homeostasis [26,27]. Silica nanoparticles are usually administered directly into the circulation by means of intravenous injection, alternatively, they can be put into circulation orally, in a way of inhalation, or direct interaction with circulating cells such as erythrocytes. Nevertheless, it is important to emphasize that our knowledge about interactions of SiNPs with red blood cells is still extremely limited [28].

The aim of this paper is to compare the biological effects of mesoporous silica nanoparticles extracted from *Urtica dioica L.* and pyrogenic silica in direct contact with normal cells (endothelial cells and red blood cells).

This study was based on the green synthesis of nanoparticles which is viewed as an emerging field of nanotechnology and seems to be extremely attractive in medicine, the pharmaceutical industry, and other sciences within this area. What is more, green synthesis of nanoparticles, which is based on plants as reducing agents, is viewed as being efficient, cost-effective, fast, and eco-friendly. To the best of our knowledge, this study presents a novel method of producing biogenic silica nanoparticles (*bio*SiO_2_) of high-purity which is obtained from stinging nettle. Therefore, in this study, we propose to analyze their biocompatibility using the in vitro cell-based approach.

These nanoparticles were analyzed with the help of scanning electron microscopy, transmission electron microscopy, and infrared spectroscopy technique ATR FTIR. Obtained TEM and SEM images reveal the aggregated, spherical shaped particles, of size varying from 6–20 nm (for both biogenic pyrogenic nanoparticles) and amorphous structure. The efficiency of the proposed method was confirmed by determining the high purity of the final product (*bio*SiO_2_) using the ATR FTIR method. We can observe the spectrum of the functional group characteristic for silica-based materials (see Figure 2).

It is important but still not unanimously decided whether silica dioxide nanoparticles are toxic or not. According to the United States Food and Drug Administration, silica is a safe material and its nanoparticles can be used in clinical research as a carrier of different compounds and biomolecules. Although experiments which were conducted using colloidal, porous, and non–porous silica have proved that this material can be used as a carrier for drugs [6], the problem of silica dioxide nanoparticles toxicity still has not been fully explained.

Interestingly, certain in vitro experiments suggest that silica nanoparticles have a detrimental impact on cells, but it depends on both the type of cells that are studied and the size of SiNPs used. It has been proved that nanoparticles are able to damage a cell membrane e.g., in a way of producing reactive forms of oxygen, increasing the production of maleic aldehyde, decreasing the level of glutathione, as well as inducting antioxidative enzymes [29]. Moreover, Yu et al. [30] analyzed the impact witch nanoparticles have on various cells (cancerous epithelial cells and macrophages). The obtained results proved that whereas epithelial cells showed considerable resistance towards nanoparticles, in the case of macrophages, their resistance depended upon the surface charge. Another study of endothelial cells conducted by Napierska et al. [31] showed that the smaller monodisperse amorphous SiO_2_ are, the more toxic they become. Yet another analysis of silica dioxide nanoparticles toxicity conducted by Murugadoss et al. [32] showed that all types of SiNPs are cytotoxic. In comparison to the cells which were not treated with silica dioxide nanoparticles, significant toxicity was observed for a concentration of 25 µg/mL. Moreover, both the size and concentration of nanoparticles had an impact on inducing oxidative stress and the process of apoptosis. Furthermore, it was proved that certain types of silica nanoparticles show genotoxicity. Colloidal silica nanoparticles may be responsible for platelet aggregation and endothelium dysfunction which, in turn, may lead to vascular thrombosis and atherosclerosis.

The studies presented in this paper clearly indicate that the usage of silicon nanoparticles leads to a decrease in the survival rate of cells which was proved using spectrophotometric (MTT) and fluorometric methods (Hoechst 33258). Moreover, while testing the impact of both types of nanoparticles (*bio*SiO_2_ and *pyr*SiO_2_) on endothelium cells, the level of RFT increased, which may induce both apoptosis and necrosis. The obtained results show that the safest concentration of the nanoparticles equals 50 μg/mL. Moreover, a substantial difference in the survival rate of cells is observed which is caused not only by the doses used in the test, but also by the type of nanoparticles, namely either *bio* or *pyr*. Furthermore, significant changes between biogenic and pyrogenic nanoparticles are observed at the highest concentrations. In this paper, statistically important changes, while compared with control, are presented. The study of the impact of silicon nanoparticles on the level of reactive forms of oxygen proves that the concentration of 50 μg/mL is safe for endothelium cells. Statistically significant changes are observed in higher concentrations. The study conducted by Yang et al. [33] in which human normal hepatocytes HL-7702 cells were treated with silica particles with a diameter of 60 nm proved that the concentration of 50 μg/mL has only a slight impact on the survival rate of cells, but is responsible for inducing processes of apoptosis and damaging DNA. This proves that the types of nanoparticles and cell lines used in tests are of the highest importance.

Moreover, the impact of nanoparticles on changes observed in cells morphology was studied. In order to evaluate the toxicity of silica nanoparticles on endothelial cells, cellular morphology and cell viability were determined. To achieve this goal, HMEC-1 cells were exposed to biogenic and pyrogenic silica nanoparticles in three incubation periods (24, 48, and 72 h). On the one hand, increasing concentration led to morphological changes in cells, on the other hand, irregular shape and cellular shrinkage were noticed when a reduction in cell density was observed (Figure 5 and Figure 6). Compared to the control group, the cell density in samples which were treated with 50 μg/mL of pyrogenic and biogenic nanoparticles was significantly reduced after 24, 48 and 72 h of exposure. Furthermore, the observed changes in cellular morphology were directly reflected in cell viability. Whereas viability of HMEC-1 cells induced by silica nanoparticles showed no significant change after 24 h, in a longer period of time, namely after 72 h, cell survival rate in a sample treated with 50 μg/mL of nanoparticles decreased to about 80%—a result which was lower than that of control. Also Duan et al., [26] in their study proved that compared to control group, the viability of the endothelial cells in a group was treated with 100 μg/mL of pyrogenic and biogenic nanoparticles was reduced after 24 h of incubation. Moreover, changes in cellular morphology were observed and recorded using both optical microscopy and TEM images.

Currently, there are numerous available types of SiO_2_ nanoparticles, which are different in size, shape as well as both the presence and size of pores. Many of them are used in biomedicine as prospective carriers of different compounds, in particular drugs. Usually, they are administrated intravenously, therefore, it is important to examine their impact on erythrocytes, which are the dominant cells in blood, as well as on endothelial cells that cover blood vessels.

In this study, the impact of SiO_2_ nanoparticles on erythrocytes in a body was investigated. The particles used in the tests were of a spherical shape and size between 6–20 nm. They were diversified in size and degree of porosity. The incubation period was 2 h and 24 h and the temperature, in which erythrocytes were tested using SiO_2_ nanoparticles, was either 37 °C. The conducted experiments showed that SiO_2_ nanoparticles are able to induce hemolysis in erythrocytes to a degree which is both concentration and time-dependent. Whereas after a 2 h incubation period at the temperature 37 °C and in the concentration 200 µg/mL, hemolysis was observed at the level 70.75%, respectively, after a 24 h incubation period, hemolysis was even higher. Similar results regarding the relationship between the degree of hemolysis and the concentration of the used SiO_2_ nanoparticles were obtained by Nemmar et al. [28]. In their experiment, after a 30 min incubation period at room temperature and for concentration of 1 µg/mL, the degree of hemolysis was significantly lower than after a 2 h incubation period. Their results correspond with the results obtain in our study and prove that SiO_2_ nanoparticles show toxicity towards RBCs and are responsible for their time and concentration-dependent decomposition. Very interesting and surprising results were obtained in relation to *bio*SiO2 nanoparticles, for which the level of hemolysis after 2 h and 24 h of incubation significantly less than *pyr*SiO_2_. As shown in Figure 10 these nanoparticles are much safer for red blood cells and non-toxic in the highest concentrations.

As it was mentioned above, SiO_2_ nanoparticles are responsible for deformation effects observed within erythrocyte membranes, an observation which was also proved in this study. On the basis of numerous experiments conducted throughout decades, it is commonly known that the shape of erythrocytes may be modified either using different modifiers [34], or as a consequence of pathological changes in a body [35]. Due to multiple studies and scientific theories, we are able to determine, on the basis of RBCs shapes, particular places of concentration of the tested compounds in a lipid bilayer of the erythrocyte membrane. The used SiO_2_ nanoparticles were responsible for modification in the shape of RBC, namely from discocytes to echinocytes and even stomatocytes. The number of modifications observed in erythrocytes was time and concentration-dependent. When a higher concentration of *pyr*SiO_2_ nanoparticles was used, an increasing number of echinocytes was observed. Moreover, RBCs began to aggregate. Regarding *bio*SiO_2_, whereas after a 2 h incubation period both stomatocytes and echinocytes were observed, after a 24 h incubation period echinocytes were dominant. Similar results were obtained by Joglekar et al. (2013) [36] and Tsamesidis et al. [37]. In their experiments, as a result of interaction with the spherical nanoparticles, erythrocytes were modified into echinocytes. Moreover, the above experiment showed that at a concentration of 100 µg/mL, a cell membrane is not deformed at the place of connection with a nanoparticle. Deformation was observed only under the concentration of 500 µg/mL. Furthermore, nanoparticles were not absorbed by erythrocytes, regardless of their particular shapes. In addition, insignificant speculation and deformation of membrane for the concentrations 20 and 50 µg/mL were observed. In the study conducted by Jiang et al. [38], these RBCs which were incubated using SiNPs had an irregular shape which resembles swelling. Moreover, cell membrane fragments were noticed. When the number of erythrocytes in the field of view was counted, it was observed that when RBCs are treated with SiO_2_ nanoparticles the total number of erythrocytes is lower, a fact which may be responsible for hemolysis. In contrast to the results obtained in this paper, [39] Zhao et al. showed that smaller particles of MCM-41 type are able to adhere to the surface of the membrane and do not cause deformation. Besides, when compared with control, they maintained a typical for erythrocytes biconcave shape. Bigger nanoparticles (SBA-15) were responsible for a local deformation of a membrane, which, in turn, led to the encapsulation of erythrocyte and the formation of echinocyte. As a result of the above process, the coefficient of the surface area was reduced, which might be the reason for hemolysis in these cells.

Certain biomaterials, when considered in vivo tests, do not show a significant increase in hemolysis. However, they are able to exert impact on the structure of the RBC membrane which, under in vivo conditions, may lead to damage. Normal erythrocytes, in hypotonic solution and under concentrations similar to isotonic, show resistance to hemolysis due to a possible increase in their volume. This is possible for a non-deformed cell thanks to a proportion between the volume ratio and the size. When disproportion is noticed, a lower degree in osmotic resistance is observed. In this paper, the study focused on the degree of osmotic resistance of erythrocytes which were impacted by *pyr*SiO_2_ showed that nanoparticles at non-lytic concentrations (50 µg/mL) are able to reduce the degree of cells osmotic resistance after a 2 h incubation periods. Furthermore, a 2 h incubation period of nanoparticles SiO_2_ and erythrocytes in concentrations of 20 µg/mL and 50 µg/mL was responsible for an increase in the degree of osmotic resistance. However, the differences between the percent of hemolysis of control RBC and the concentrations of 20 µg/mL and 50 µg/mL were statistically insignificant. The studies of Zhao et al. [39] proved that SiO_2_ nanoparticles are responsible for the reduction in size compared to the volume in erythrocytes, which is the main reason for a decrease in the degree of osmotic resistance observed for *pyr*SiO_2._

Considering all these abovementioned experiments, it may be stated that silica dioxide nanoparticles show no toxicity towards endothelial cells and erythrocytes, but only within a certain range of concentrations. The results of these studies prove that this safe concentration level for *bio*SiO_2_ nanoparticles and cells equals 50 μg/mL. Although bio-silica dioxide nanoparticles are a promising material that may be used in many applications in biology and medicine, further studies are required in order to prove both their safety and efficacy and to determine under which conditions and concentrations they are not toxic to the human body.

## 4. Materials and Methods

### 4.1. Silica Nanoparticles

For this study, we selected a type of plant which has a high content of biogenic silica, namely nettle *(Urtica dioica L.)*. Stinging nettle *(Urtica dioica L.)* samples were purchased from BiFIX Wojciech Piasecki Sp. j. (Górki Małe, Poland). Silicon dioxide nanoparticles were isolated from stems and leaves contained in the BiFix tea. In this process, a concentrated nitric acid was used. To remove the organic phase, the sample was weighed in a reaction vessel and nitric acid was added. Subsequently, the vessel was sealed and placed in the reactor. An ERTEC (Wrocław, Poland) microwave reactor was used in order to prepare a nanosilica powder. Decomposition was carried out for 20 min at 190 °C and under the pressure of 45 bar. The mineralized samples were transferred to PE containers and diluted. The samples of nanoparticles were rinsed with deionized water in order to obtain a neutral pH. In the following step, the white silica powder was dried in a laboratory heating oven at 100 °C for 12 h. Anhydrous ethanol, hydrochloric acid, nitric acid (Penta Chemicals, Prague, Czech republic), and deionized filtered water were used in all purification processes in this study. The yield of the prepared extraction was around 15% [40].

#### 4.1.1. Transmission Electron Microscopy (TEM)

Transmission Electron Microscopy (TEM) was used to determine the shape and size of nanoparticles. Powder samples were suspended in anhydrous ethanol and ultra-sonified using an ultrasonic homogenizer (20% amplitude). The prepared suspension was centrifuged for 5 min at 6000 rpm. Supernatants from each sample were dropped on a copper grid with holey carbon film and dried on air. Transmission Electron Microscopy analyses were conducted using a JEM 2010F device (JEOL, Akishima, Japan) at 160 kV of accelerating voltage.

#### 4.1.2. Scanning Electron Microscopy (SEM)

The silicon dioxide nanoparticles were characterized by a scanning electron microscope (SEM). Samples of silica powder were placed on the carbon tape on the holder and the images were performed using a FE SEM ULTRA PLUS device (ZEISS, Gina, Germany).

#### 4.1.3. Fourier Transform Infrared Spectroscopy (FTIR)

Infrared spectroscopy technique Attenuated Total Reflectance (ATR) FTIR was used for the qualitative characterization of both *bio*SiO_2_ and *pyro*SiO_2_ samples. The characteristic functional group’s presence has been confirmed and verified using VERTEX 70 spectrometer (Bruker, Mannheim, Germany). The investigation was performed over a wide wavenumber range 4000–400 cm^−1^ (resolution of 0.5 cm^−1^).

#### 4.1.4. Hydrodynamic Particle Size Distribution

The size of particles was determined by a Zetasizer Nano ZS instrument (Malvern Instruments Ltd., Malvern, UK) using a non-invasive back scattering (NIBS) method. For analysis 10 mg of each samples have been dispersed in 12 mL of deionized water using sonication at 60 kHz with a Polsonic Sonic-3 (Warsaw, Poland) for 10 min. The measurements of particle size were 30 times repeated for each sample of examined silica powders.

#### 4.1.5. Thermogravimetric Analysis (TGA/DTG)

The qualitative characterization of the both obtained materials have been determined by thermogravimetric analysis (TGA) using a Jupiter STA 449 F3 analyzer (Netzsch, Selb, Germany). Samples weighing approximately 10 mg were placed onto thermobalance and heated from 30 to 1000 °C at a heating rate of 10 °C/min in a nitrogen atmosphere. Additionally, DTG curve was plotted to better visualize the thermal decomposition process.

### 4.2. Cell Culture

HMEC-1, namely a human dermal microvascular endothelial cell line (ATCC CRL 3243), was purchased from American Type Culture Collection (ATTC, Dziekanów Leśny, Poland). The cells were cultured in MCDB 131 medium, containing 10% fetal bovine serum (FBS), 10 mM L-glutamine, 1 μg/mL hydrocortisone, 1% penicillin/streptomycin, and 10 ng/mL epidermal growth factor (EGF) purchased either from Gibco (Warsaw, Poland) or Sigma Aldrich (Poznań, Poland), under 5% CO_2_ in plastic flasks at 37 °C. The cells density for seeding was chosen in order to assure the logarithmic cells growth until analysis.

#### 4.2.1. Optical Microscopy

Cells (HMEC-1) were seeded on 6-well plates (250 000 cells per well) and cultured for 12–24 h. After that time, silica nanoparticles (biogenic and pyrogenic) were added at the concentration of 0, 20, 50, 100, and 200 μg/mL and the incubation was continued for 24, 48, and 72 h. After that time, the cells were washed twice in Dulbecco’s Phosphate Buffered Saline (DPBS) and fixed with ice-cold 100% methanol for 10 min at −20 °C. Subsequently, the cells were washed gently 3 times in DPBS. The cells were moved off the freezer to room temperature, subsequently kept in 0.5% crystal violet solution in 25% methanol, and incubated for 10 min. The crystal violet was removed and the cells were washed in water several times until the dye stopped coming off. The samples with cells were allowed to dry at room temperature. In the next step, the morphology of cells was analyzed using an Olympus CKX53 (Olympus, Tokyo, Japan), 100×.

#### 4.2.2. Cytotoxicity Test

The effect of the SiO_2_ nanoparticles on the proliferation of HMEC-1 was estimated by the ability of the cells to reduce the tetrazolium dye 3-(4,5-dimethylthiazol-2-yl)-2,5-diphenyltetrazolium bromide (MTT) to its insoluble formazan. The tested cells were seeded on 96-well plates (5000 cells per well) and cultured for 12–24 h. After that time, silica nanoparticles were added at appropriate concentrations (0–200 μg/mL) and the incubation was continued for the following 24, 48, and 72 h. Subsequently, the monolayers of cells were rinsed in a Hanks’ balanced salt solution (HBSS), added together with fresh medium and 20 μL of MTT solution (5 mg/mL). After 2 h of incubation, the medium was removed, and the formed formazan crystals were dissolved in DMSO. The absorbance of the formazan was read at the level of 570 nm (its values are proportional to the number of viable cells in a sample).

#### 4.2.3. Assay of Cell Survival Based on DNA Content

In order to analyze DNA content (cytotoxicity test), the fluorescent probe Hoechst 33,258 was used. Cells, seeded on a 96-well black plate at the density of 5000 cells per well, were grown under 5% CO_2_ at 37 °C, treated with silica nanoparticles (0–200 μg/mL), and incubated for 24, 48, and 72 h. After that period, the medium was removed by gentle aspiration, and the cells were rinsed twice with HBSS and the plate was frozen at −70 °C. Thawing at room temperature (RT) was followed by adding 100 μL of deionized water per well and freezing once again at −70 °C. After subsequent thawing, 100 μL per well HVAB (0.5% Hoechst 33,258 in TNE buffer (2 M NaCl, 1 mM EDTA, 10 mM Tris–HCl, pH = 7.4)) was added. The plate was shaken gently, incubated at RT in the dark for 15 min and the level of fluorescence was read at 355/460 nm.

#### 4.2.4. Determination of ROS Production

Reactive oxygen species production was estimated in human dermal microvascular endothelial cells seeded on 96-well black plates (5000 cells per well) and cultured for 24 h. After that time, silica nanoparticles were added at appropriate concentrations and the incubation was continued for another 24, 48, and 72 h at 37 °C under 5% CO_2_. Subsequently, the cell monolayers were rinsed with HBSS, and in the following step fluorogenic probe, 5 μM 2′,7′- dichlorodihydrofluorescein diacetate (H_2_DCF-DA) in HBSS was added. Nonfluorescent H_2_DCF-DA is hydrolyzed intracellularly to form 2′,7′- dichlorodihydrofluorescein, which is oxidized by ROS to a fluorescent 2′,7′-dichlorofluorescein. After 2 h incubation in the dark at 37 °C under 5% CO_2_, the fluorescence was read at λ_ex_ = 485 nm and λ_em_ = 538 nm.

Next, DNA content was determined. For this purpose, after measurements of ROS, the cells were frozen at −70 °C. After that, the cells were thawed, 100 μL of distilled water per well was added and the cells were re-frozen. Afterward, the cells were thawed again. In next step, 50 μL of 15 μg/mL of ribonuclease A was added to each well and incubated (30 min in the dark at 37 °C). Further away, 50 μL of 10 μM propidium iodide in deionized water was added to the cells and incubated for 15 min in the same conditions. Finally, the fluorescence intensity of PI was measured at λ_ex_ = 355 nm and λ_em_ = 620 nm. The rate of ROS generation was calculated according to the following formula:

Rate of ROS = intensity of H_2_DCF-DA fluorescence/intensity of PI fluorescence

The value of fluorescence of control samples was assumed as 100%.

### 4.3. Erythrocytes

The studies were also conducted using pig red blood cells. The choice of pig erythrocytes was based on the fact that these cells are the closest to the human erythrocyte, and the blood was readily available. The erythrocytes were obtained from fresh, heparinized pig blood and for washing the erythrocytes, an isotonic phosphate solution of pH 7.4 was used.

#### 4.3.1. Hemolysis of Erythrocytes

The haemolytic assay was prepared following the method described by Pruchnik et al. [41]. Full blood (fresh, heparinized pig blood) was centrifuged (2500 rpm, 3 min, 4 °C) in order to remove plasma and leucocytes, and next red blood cells (RBCs) were washed three times with cold phosphate-buffered saline isotonic solution (pH 7.4) PBS—310 mOsm. The test sample (1 mL) contained an appropriate volume of phosphate-buffered solution, the silica nanoparticles compounds, and erythrocytes at a final hematocrit of 1.2%. The hemolytic activity of the compounds was determined for concentrations from 0 to 200 µg/mL after 2 and 24 h of incubation at 37 °C. After that time, 2 mL of phosphate buffer (pH 7.4) were added and the samples were centrifuged (2500 rpm, 15 min) at RT (room temperature). The supernatant was assayed for the hemoglobin content using a spectrophotometer on the UV–Vis spectrophotometer (Specord 40, Analytik Jena, Jena, Germany) at 540 nm wavelength. The hemoglobin concentration in the supernatant, expressed as a percentage of the hemoglobin concentration in the supernatant of totally haemolysed cells, was assumed as the measure of the extent of hemolysis. Samples with total hemolysis (100%) were prepared by adding deionized water (dH_2_O) to the negative control samples.

#### 4.3.2. Osmotic Resistance Assay Was Performed on Fresh

The osmotic resistance assay was performed using fresh pig blood prepared in the same way as during hemolysis. Subsequently, 1.2% red cell suspension containing silica nanoparticles of 20 and 50 μg/mL concentration was prepared in 0.9% NaCl and left for 2 and 24 h at 37 °C with continuous stirring. After this incubation, the suspension of erythrocytes was centrifuged for 15 min at room temperature in order to remove cells from SiO_2_. From the cell sediment, 100 µl samples of the extract-modified cells were removed and suspended in test tubes containing NaCl solutions at the 0.5–0.86% concentration and an isotonic (0.9%) NaCl solution. In solutions, the same concentrations were also suspended in unmodified red blood cells which constituted control for osmotic resistance determinations. Subsequently, the suspension was stirred and centrifuged under the above-described conditions. Next, the percentage of hemolysis was measured with a spectrophotometer at λ = 540 nm wavelength and, on the basis of the results obtained, the relation between the percentage of hemolysis and the NaCl concentration in the solution was determined. This test proves that whenever a determined sodium chloride concentration is higher than that of control cells, the osmotic resistance of the erythrocytes is regarded to be lower, and vice versa [42].

#### 4.3.3. The Microscopic Studies of Erythrocyte Shapes

The impact of the extract on the shape of erythrocytes was determined using an optical microscope. The red cells, separated from plasma, were washed three times in saline solution and suspended in the same solution but containing 20 and 50 μg/mL of the silica nanoparticles. Haematocrit of the erythrocytes in the modified solution was determined at the level of 1.2% and the modification lasting 2 and 24 h at 37 °C. After modification, erythrocytes were fixed with a 0.25% solution of glutaraldehyde. After that erythrocytes were observed under an optical microscope (Eclipse E200, Nikon, Wrocław, Poland city) equipped with a digital camera. The obtained photographs made it possible to count erythrocytes of various shapes, and then the percent share of the three basic forms (discocytes, echinocytes and stomatocytes) in a population (3 times) of ca. 300 cells was determined. Individual forms of the erythrocyte cells were ascribed to morphological indices according to the Bessis scale [43].

Changes in shape of red blood cells were also observed using a scanning electron microscope. To meet this goal, red blood cells were prepared in a similar way to studies in which an optical microscope was used, as it was described above. In the following step, erythrocytes were fixed with a 4% solution of glutaraldehyde diluted in a 0.2 M phosphate buffer (pH 7.4) over a period of 8 h. Next, the analyzed material was flushed in a 0.2 M phosphate buffer (pH 7.4) over a period of 24 h. Furthermore, samples were put into 2% osmium tetroxide (OsO_4_) over a period of 2 h and flushed with distilled water over another 30 min. Finally, samples were deoxidized in the series of alcohols which were concentrated as follows: 50%, 70%, 80%, 90%, and three times 100% and over a period of 15 min in each of these concentrations. After that, samples were placed on coverglass, sprinkled with hexamethyldisilazane, and dried in the air. After all these steps, the material tested was fixed to a subject table of a microscope and sprayed using a vacuum sprayer with both spectrally pure carbon and silver (Ag). Samples were analyzed using a Tesla–300 scanning electron microscope (SEM, Tokyo, Japan) as it is illustrated in Figure 12.

### 4.4. Statistical Analysis

All data are mean and SD from at least three independent experiments. Statistical evaluation of differences was made using the ANOVA I and Tukey’s post hoc test, at significance levels of *p <* 0.01 (*).

## 5. Conclusions

In this study, an attempt was made to synthesize *bio*SiO_2_ from *Urtica dioica L*. and to analyze its biocompatible properties using in vitro cell-based approach. Furthermore, the obtained results were compared with these obtained with the use of a commercially available type, namely pyrogenic silica nanoparticles. The size of both forms of amorphous silica nanoparticles (both *bio* and *pyr*) was no larger than 20 nm, as confirmed using different analytical and microscopic techniques. This study paves a way for synthesizing SiNPs from plants which are able to store such a silica material which parameters are extremely similar to synthetic products. As presented, biogenic nanoparticle (in case of human dermal microvascular endothelial cell line) have either similar or (in case of the red blood cells) even better effect on cells. We conclude that SiO_2_ nanoparticles are safe for normal cells but within an appropriate dosage. Therefore, it is impossible to state that bio nanoparticles are leading in nanotechnology due to the fact that more experiments must be conducted. Our method of synthesis is fully compliant with all the principles of green chemistry and is environmentally friendly. Furthermore, it is also extremely promising as far as a broad range of processes and applications in the biomedical and pharmaceutical industries are concerned.

## Figures and Tables

**Table 1:** The level of osmotic resistance in relation to the concentration of psychological saline (IC_50_) which is responsible for 50% hemolysis of erythrocytes which were modified by SiO_2_ nanoparticles.

IC_50_ (μg/mL)		
Time Incubation	2 h	24 h
Control	0.68 ± 0.018%	0.77 ± 0.021%
*bio*SiO_2_ 20 μg/mL	0.63 ± 0.015%	0.60 ± 0.040%
*bio*SiO_2_ 50 μg/mL	0.63 ± 0.014%	0.65 ± 0.037%
*pyr*SiO_2_ 20 μg/mL	0.68 ± 0.020%	-
*pyr*SiO_2_ 50 μg/mL	0.72 ± 0.011%	-

## Data Availability

The data presented in this study are available in supplementary material.

## Supplementary Materials

The following are available online. Figure S1: TEM analysis images indicate that *bio*SiO_2_ presents a spherical shape of single nanoparticles.

## Sample Availability

Samples of the compounds are not available from the authors.
