# Supplementary material for: Are Biogenic and Pyrogenic Mesoporous SiO2 Nanoparticles Safe for Normal Cells?
Source: Molecules. 2021 Mar 6;26(5):1427. doi: 10.3390/molecules26051427 (PMC7961954; doi:10.3390/molecules26051427)
Supplement: Supplementary file 1 [file molecules-26-01427-s001.pdf]

Supplementary materials

Are biogenic and pyrogenic mesoporous SiO_2 nanoparticles safe for normal cells?

Solarska-Ściuk K[1], Adach K[2], Cyboran-Mikołajczyk S[1], Bonarska-Kujawa D[1], Rusak A[3], Cwynar-Zając Ł[3], Machałowski T[4], Jesionowski T[4], Grzywacz K[1], Fijalkowski M[2].

[1]Department of Physics and Biophysics, Wrocław University of Environmental and Life Sciences, Wrocław, Poland

[2]Department of Advanced Materials, Institute for Nanomaterials, Advanced Technologies and Innovation, Technical University of Liberec, Czech Republic

[3]Department of Histology and Embryology, Medical University of Wrocław, Wrocław, Poland

[4]Institute of Chemical Technology and Engineering, Faculty of Chemical Technology, Poznan University of Technology, Poznan, Poland

Corresponding author: solarskakatarzyna.biol@gmail.com, katarzyna.solarska-sciuk@upwr.edu.pl

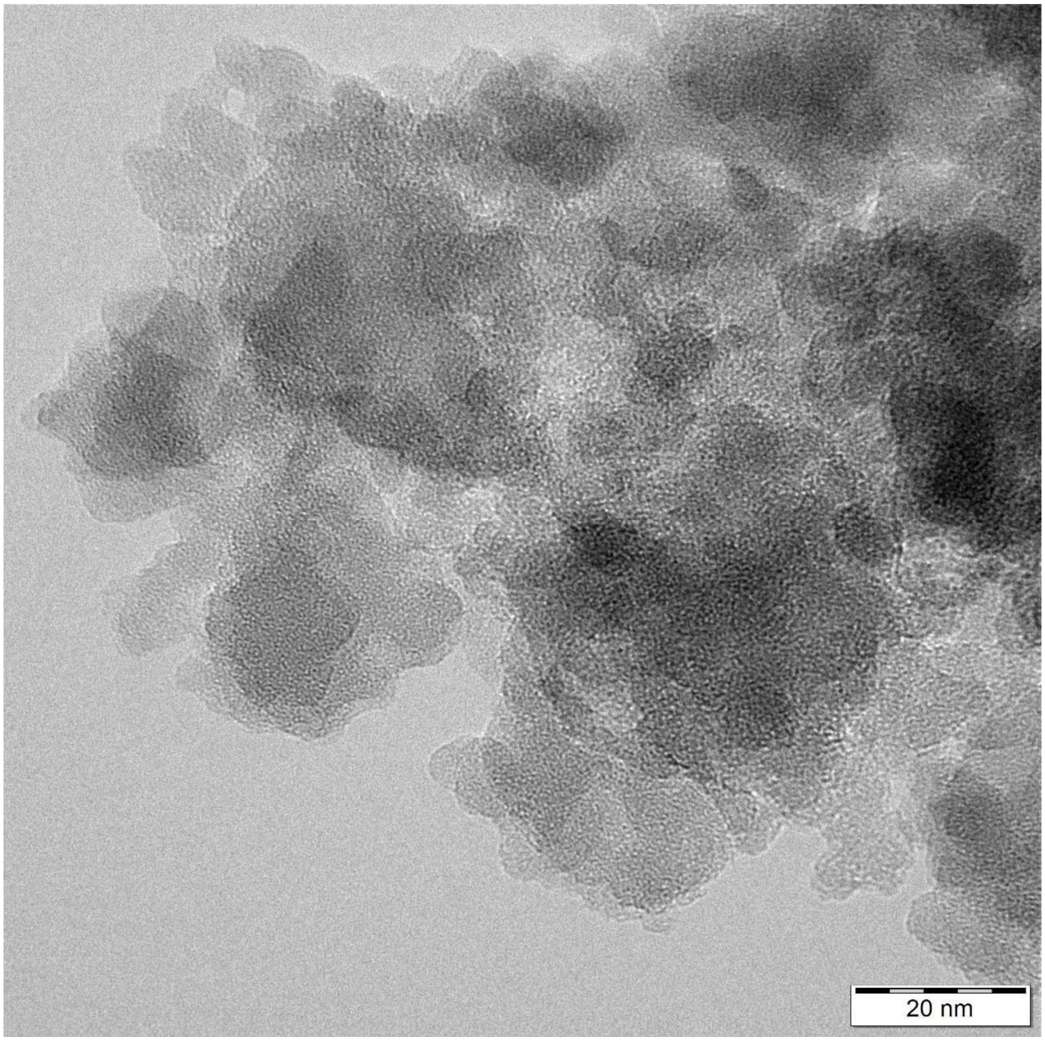

Figure S1. TEM analysis results.
